# Comparative Effectiveness of Different Immobilization Techniques in Managing Reduction of Acute Anterior Shoulder Dislocation: A Retrospective Cohort Study

**DOI:** 10.1007/s43465-025-01497-0

**Published:** 2025-08-19

**Authors:** Yao He, Liangqian Wang, Wei Bao, Yuandong Zhou, Denghua Liu, Denghua Huang, Yuanjun Fan, Chuanbo Li, Yongjun Hu

**Affiliations:** 1 Department of Orthopedics, Banan Hospital of Chongqing Medical University, No. 659, Yunan Avenue, Banan District, Chongqing, China; 2 Department of Orthopedics, The Central Hospital Affiliated to Chongqing University of Technology, Chongqing, China

**Keywords:** Shoulder dislocation, Reduction, Immobilization, Rehabilitation, Computed tomography

## Abstract

**Purpose:**

The treatment of primary traumatic anterior shoulder dislocation varies widely. However, recent basic science and clinical data do not unify the specific immobilization methods. We aimed to compare the effectiveness of immobilization in internal rotation, external rotation and external rotation combined abduction after primary anterior dislocation of the shoulder.

**Methods:**

A total of 165 patients (80 males and 85 females, average age, 61 years, range 18–73 years) with the diagnosis of acute primary traumatic anterior shoulder dislocation were immobilized at in internal rotation (IR, *n* = 62), external rotation (ER, *n* = 53), external rotation combined abduction (ERAb, *n* = 50) after manipulative reduction. The mean follow-up period was 23.85 months (range 16–28 months). Patients received rehabilitation program immediately after immobilization. We assessed functionality by constant-Murley score (CMS), American Shoulder and Elbow Surgeons Scale (ASES) and stability by Western Ontario Shoulder Instability Index (WOSI) in *week 6, months 3, 6, 12, and 24*. Otherwise, the measured humeral upward distance (HUD) and humeral forward distance (HFD) on Computed Tomography (CT) of the shoulder were determined in day 1 and sixth weeks after immobilization.

**Results:**

for HUD and HFD, there were statistically significant differences between shoulder joint immobilization at 1 day and 6 weeks within each group (*P* < 0.001), and there were all significantly bigger than in the contralateral side on the day 1 of immobilization (*P* < 0.001), but after 6 weeks of shoulder joint immobilization, although slight differences existed between the affected and non-affected side in each group, none of these varieties reached significance (*P* > 0.05). Intragroup comparisons of the constant-Murley score (CMS), American Shoulder and Elbow Surgeons Scale (ASES) and Western Ontario Shoulder Instability Index (WOSI) in the different time periods revealed significant differences (*P* < 0.001). Furthermore, upon release of immobilization at the 6-week postoperative interval, the ERAb group demonstrated superior early phase mobility and functional recovery compared to the other two cohorts. However, longitudinal evaluation revealed no statistically significant differences in functional outcomes among the groups after the 3-month follow-up assessment.

**Conclusions:**

Internal rotation, external rotation, and external rotation combined abduction immobilization are effective methods for reducing postoperative complications of acute anterior shoulder dislocation and we recommend the external rotation combined abduction immobilization approach to promote early recovery of shoulder function.

## Introduction

The shoulder possesses the highest degree of movement as it is a ball-and-socket joint known as the glenohumeral joint. Given its flexible structure, it is the joint most prone to instability within the human body [1]. Shoulder dislocation is a frequent and painful injury. The patient’s progress following non-surgical treatment has been extensively studied, with a focus on the notably high rate of repeated instability. Recurrence rates for these patients have been estimated to fall anywhere between 17% and 96% [2–4]. A dislocation can occur in four directions: anterior, posterior, inferior, or superior. However, the anterior dislocation is notably the most prevalent, responsible for up to 98% of all reported cases [5]. The anterior traumatic dislocation constitutes 58.8% of all shoulder dislocations [4] and is correlated with ligament disruption, fractures, rotator cuff injuries, and neurologic involvement [6, 7], consequently, pain, disability, and apprehension are present during some shoulder movements [8]. Therefore, acute shoulder dislocation is a medical emergency that necessitates immediate repositioning. Numerous methods have been proposed for treating shoulder dislocations. The goal of effective treatment is to restore a fully functional, painless, and stable joint in the shoulder [9].

Shoulder dislocation often accompanies injuries to the labrum and rotator cuff. The conventional approach for healing anterior shoulder dislocation is to rest the arm in an internally rotated position for a period of 3–6 weeks after reduction. This is followed by rehabilitation therapy. However, the effectiveness of this treatment is not well-established. Chronic shoulder instability may lead to pain. This discomfort is usually position-dependent and can broadly impact one’s overall quality of life [10–12]. Biomechanical research has determined that, in case of glenoid labrum tear, there is not any contact on the glenolabral surface during internal rotation. The contact is minimal when in a neutral position, yet it maximizes at 45 degrees of external rotation [13]. Based on a cadaveric study and Magnetic Resonance Imaging (MRI), Itoi and his team theorized that in external rotation, the anterior shoulder soft tissue would become taut. This might prevent the formation of a haematoma, thus encouraging the Bankart lesion to adhere better to the glenoid neck [14, 15]. Based on their findings, Itoi and his team proposed that immobilizing the joint in an externally rotated position could reduce the recurrence rate of dislocations. They began a study to test this theory. The study’s conclusion affirmed their hypothesis. Notably, it showed that external rotation immobilization lessened the risk of repeat dislocations compared to internal rotation immobilization [16].

In a recent analysis, Braun and colleagues [9] concluded that there is not enough evidence to determine whether immobilizing the arm in external rotation (ER) provides any benefits over internal rotation (IR). However, consideration must be given to the fact that these studies used varying angles of ER, ranging from 0° to 30°. Miller et al. [13] displayed that the interaction force between the labrum and the anterior glenoid increases with ER, reaching its peak beyond 45° of ER. Furthermore, through a controlled arthroscopic study. Hart and Kelly [17] found that the optimal in vivo position for the labrum can be achieved at 60° of external rotation coupled with an additional 30° of abduction. In a subsequent study focusing on biomechanics. Itoi et al. [18] demonstrated that elevating the arm during immobilization in ER enhances the reduction of the Bankart lesion. However, there are relatively few related studies. While younger patients dominate dislocation studies, elderly populations face unique rehabilitation challenges; our cohort addresses this evidence gap.

Moreover, prior research seldom assessed immobilization techniques via imaging. Therefore, this study intended to investigate the impact of diverse immobilization methods post-reduction of the first anterior shoulder dislocation, by studying imaging and functional alterations in a retrospective cohort study. We theorized there would not be substantial variances in recurrence rates and clinical outcomes amongst the three approaches (internal rotation, external rotation, and external rotation combined abduction immobilization), all presumably yielding satisfactory results.

## Methods

### General Information

We conducted a retrospective cohort study at a Level-A tertiary teaching hospital in Chongqing, China, with approval from the Banan Hospital of Chongqing Medical University review board. The study was registered in the Chinese Clinical Trial Registry under the registration ID: ChiCTR2300075474. The study was reported in line with the criteria for Consolidated Standards of Reporting Trials (CONSORT) [19] statement and all methods were performed in accordance with the relevant guidelines and regulations. Inclusion criteria were patients who underwent their first anterior shoulder dislocation, manipulative reduction of the shoulder joint under general anaesthesia without the need for tracheal intubation, and had their shoulder joint analyzed by MRI on the week 6 and Computed Tomography (CT) on the day 1 and week 6 after reduction. The study excluded patients with a shoulder dislocation paired with a fracture of the humeral greater tubercle, follow-up MRI and showed rotator cuff tear (including full-thickness and partial tear) or bankart’s lesions (including bony bankart’s lesions), injuries to nerves or blood vessels around the shoulder joint, or unaccountable movement limitation or pain around the shoulder joint prior to the injury. We analyzed the medical records from February 2019 to February 2022 and found a total of 165 qualifying patients. These patients were categorized into three groups: the internal rotation (IR) group (*n* = 62), the external rotation (ER) group (*n* = 53), and the external rotation combined with the abduction (ERAb) group (*n* = 50), based on the method of immobilization post-reduction.

## Manipulative Reduction

Before any emergency manual reduction procedure, every patient provided their signed, written informed consent. These procedures were all carried out under intravenous analgesia, with the patients in a supine position. Prior to the reduction, each patient had an X-ray examination to confirm a first-time anterior dislocation of the shoulder joint, and to ensure there were no surrounding fractures or neurovascular injuries. A single surgeon, using the Hippocratic method, performed all of the reductions. In addition, a single therapist oversaw the rehabilitation program for each patient.

## Immobilization and Rehabilitation

After the reduction is completed, the patient’s shoulder joint is immobilized in one of the three positions based on the treating physician’s preference: internal rotation (70°), external rotation (15°), or external rotation (60°) combined with abduction (30°) for a duration of 6 weeks. Internal rotation is maintained using a forearm sling. External rotation and external rotation combined with abduction positions are secured with plaster casts. Physical rehabilitation begins during the third week of immobilization, starting with passive range of motion (ROM) exercises. During the initial 4-week period, external rotation of the shoulder when abduction exceeding 90 degrees is contraindicated. In addition, isometric contraction training is initiated immediately to strengthen stabilizing muscle groups. Patients may resume full activities only after achieving pain-free full ROM and muscle strength recovery.

## Measurement

**Fig. 1:**
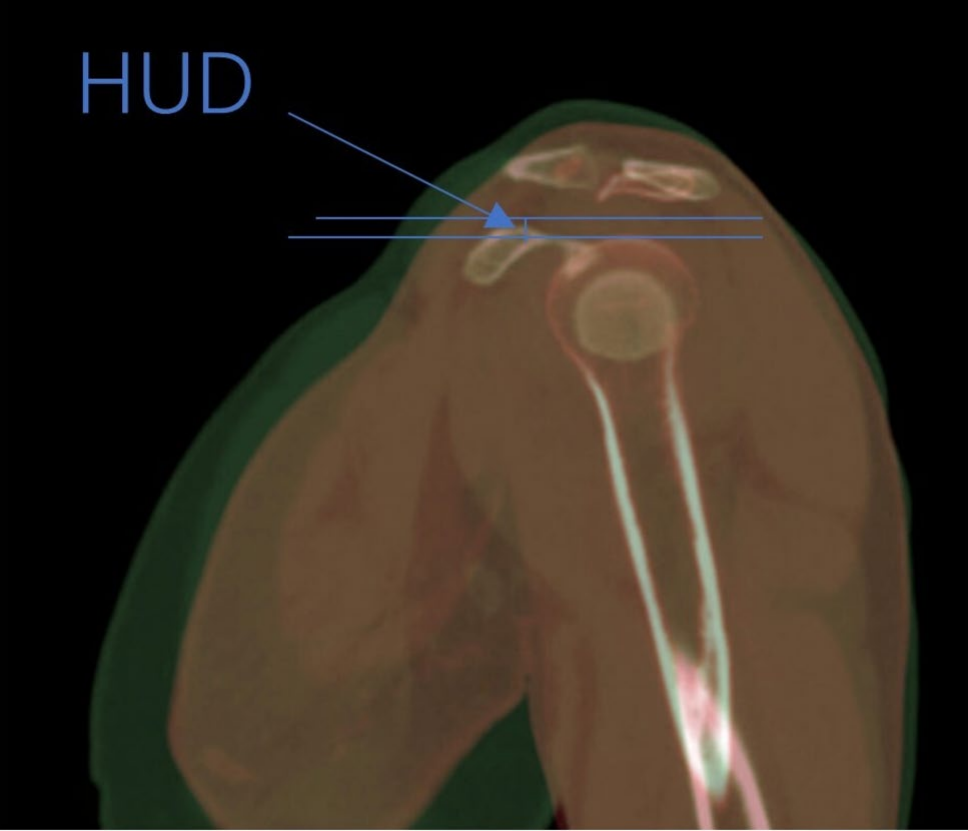
Humeral upward distance (HUD)

**Fig. 2:**
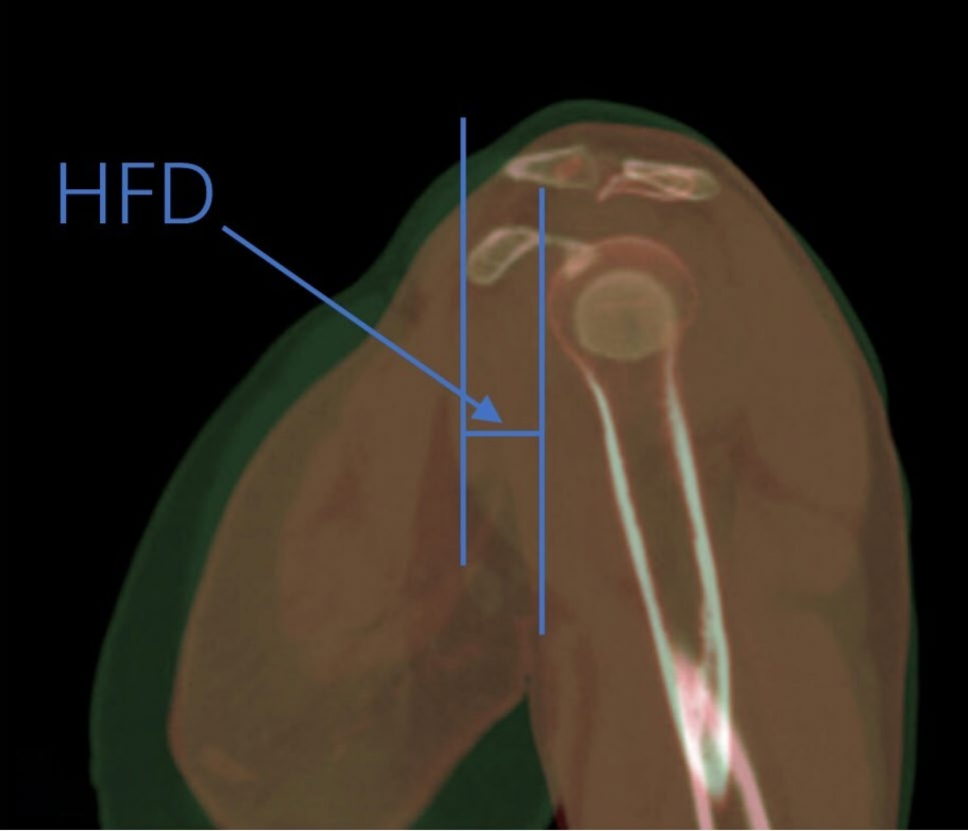
Humeral forward distance (HFD)

All measurements were conducted using an image measurement tool called Digimizer, based on Bruges, Belgium. In addition, Adobe Photoshop CS6 was employed as the professional software for image processing. All CT scans were done in lying down position and without the immobilizer. Our approach involved locating the highest points of the coracoid and the humeral head in the patient’s sagittal plane identified by CT scans. These points allowed us to gauge the humeral upward distance (HUD), described as the vertical distance between the aforementioned points, using the software tools mentioned earlier (Fig. 1). Similarly, we identified the most forward sections of the coracoid and humeral head, respectively, and computed the vertical distance between these, defining it as the humeral forward distance (HFD) (Fig. 2).

## Outcomes Evaluated

The HFD and HUD of all patients were recorded on the day 1 and then 6 weeks after immobilization. These anatomic features were manually identified by two separate readers. Following this, the previously mentioned distances were determined using the same software applications, Digimizer (Bruges, Belgium) and Adobe Photoshop CS6. Each reader measured the same parameter two times, using the average of these values for further calculations. Shoulder joint functionality on the affected side was assessed using the constant-Murley score (CMS), the American Shoulder and Elbow Surgeons Scale (ASES), and the Western Ontario Shoulder Instability Index (WOSI) at the sixth week, as well as the months 3, 6, 12 and 24 post-immobilization.

## Statistical Analysis

All assessments were conducted using SPSS 26.0 software by IBM, located in Chicago, IL, USA. Data conforming to normal distribution was represented as mean accompanied by standard deviation. A comparison of continuous variables across three groups was achieved using one-way ANOVA, while student’s *t* tests were utilized for image-based continuous data analysis. A nonparametric test allowed for intragroup comparisons. The significance threshold was established at P < 0.05. A side-to-side examination was carried out on each patient to delve into the recuperation of the joint capsule and rotator cuff following shoulder dislocation, considering varying immobilization methods. To ensure the agreement of measurements both within and among observers, intra-class correlation coefficients (ICCs) for each parameter were determined based on a two-way mixed-effects model. Should the ICCs equal or exceed 0.7, we considered this a satisfactory level of reliability.

## Results

### General Characteristics

From February 2019 to February 2022, the study encompassed 62 patients in the internal rotation group (IR), comprising 37 males and 25 females with an average age of 51 years (ranging from 18 to 73 years). There were also 53 patients in the external rotation group (ER), including 17 males and 26 females with an average age of 50 years (their ages varied from 22 to 69 years). The external rotation and abduction group (ERAb) consisted of 50 patients, encapsulating 26 males and 24 females, averaging 51 years of age (their ages spanned from 18 to 70 years). All patients underwent manual reduction, followed by regular clinical and radiological check-ups. The data collected for this study were retrospective. The comprehensive characteristics of the subjects are outlined in Table 1. There was no significant difference between groups in terms of age, sex, height, weight, body mass index (BMI), and the shoulder most commonly used.

**Table 1:** General characteristics

	IR group	ER group	ERAb group	P
No	62	53	50	
Age^b^	51.7 ± 12.5	50.2 ± 8.2	51.0 ± 10.5	0.740
Sex (F/M)^a^	25/37	26/17	24/26	0.587
Hight^b^	161.2 ± 2.8	162.1 ± 2.9	160.6 ± 2.6	0.675
Weight^b^	57.9 ± 3.3	61.2 ± 3.7	57.9 ± 3.7	0.767
BMI^b^	22.3 ± 1.1	23.3 ± 1.1	22.5 ± 1.4	0.618
Affect side (dominant/non dominant)^a^	51/11	44/9	43/7	0.859

*BMI* body mass index, *IR* internal rotation, *ER* external rotation, *ERAb* external rotation combined abduction

^a^Values expressed as number of patients

^b^Values expressed as mean ± SD

## HUD and HFD

### Differences in the Position of the Humeral Head Among the Three Immobilization Methods

Whether we’re examining HUD or HFD, notable differences were observed between 1-day and 6-week shoulder joint immobilization within each group, with a statistical significance of less than 0.001. In the internal rotation group, the HUD average was 7.04 ± 0.61 mm on day 1 and declined to 5.49 ± 1.00 mm by week 6. Concurrently, the group’s HFD average decreased from 11.64 ± 0.89 mm on day 1 to 9.44 ± 1.18 mm at the 6-week checkpoint. For those in the external rotation group, the HUD average registered at 7.01 ± 0.64 mm on day 1, then dropped to 5.36 ± 1.01 mm by week 6. Likewise, the HFD average shrunk from 11.46 ± 0.84 mm on day 1 to 9.31 ± 0.99 mm at week 6. Finally, in the external rotation combined abduction group, the day 1 average HUD was 6.81 ± 0.53 mm, decreasing to 5.51 ± 1.12 mm at week 6. The HFD average saw a drop from 11.50 ± 0.95 mm on day 1 to 9.48 ± 0.98 mm by the 6-week mark. However, no substantial difference was found when comparing each group at identical timepoints, as shown in Table 2.

**Table 2:** Differences in the position of the humeral head among the three immobilization methods

	HUD				HFD			
	1D*	6W*	△	P	1D*	6W*	△	P
IR group	7.04 ± 0.61	5.49 ± 1.00	1.55 ± 1.24	< 0.001	11.64 ± 0.89	9.44 ± 1.18	2.20 ± 1.44	< 0.001
ER group	7.01 ± 0.64	5.36 ± 1.01	1.65 ± 1.26	< 0.001	11.46 ± 0.84	9.31 ± 0.99	2.14 ± 1.41	< 0.001
ERAb group	6.87 ± 0.52	5.51 ± 1.12	1.36 ± 1.24	< 0.001	11.50 ± 0.95	9.48 ± 0.98	2.02 ± 1.58	< 0.001
P	0.283	0.748	0.502		0.52	0.712	0.815	

*HUD* humeral upward distance of the affected shoulder, *HFD* humeral forward distance of the affected shoulder, *IR* internal rotation, *ER* external rotation, *ERAb* external rotation combined abduction

△: The difference between the values from week 6 and day 1 of the same group

^*^Values were presented as means ± SDs

### Side-to-Side Differences of the Humeral Head Position Among the Three Methods

Within these three groups, the HUD on the affected side (7.04 ± 0.61 mm; 7.01 ± 0.64 mm; 6.81 ± 0.53 mm) was significantly larger than on the unaffected side (5.43 ± 0.84 mm; 5.33 ± 0.88 mm; 5.38 ± 0.84 mm, P = 0.0054) during the day 1 of immobilization. Simultaneously, a notable disparity was present in the HFD measurements (affected side: 11.64 ± 0.89 mm, 11.46 ± 0.84 mm, 11.50 ± 0.95 mm, unaffected side: 9.47 ± 0.89 mm, 9.49 ± 0.85 mm, 9.53 ± 0.81 mm, P < 0.001). Relative to the normal side, the affected humeral head had moved upward and forward, a shift that was also significant. Nevertheless, after 6 weeks of shoulder joint immobilization, while minor disparities existed between the affected and unaffected sides in each group, none of these changes were statistically significant (Table 3).

**Table 3:** Side-to-side differences of the humeral head position among the three methods

	1D						6W					
	HUD*	HUD’*	P	HFD*	HFD’*	P	HUD*	HUD’*	P	HFD*	HFD’*	P
IR group	7.04 ± 0.61	5.43 ± 0.84	< 0.001	11.64 ± 0.89	9.47 ± 0.89	< 0.001	5.49 ± 1.00	5.48 ± 0.94	0.965	9.44 ± 1.18	9.50 ± 1.17	0.791
ER group	7.01 ± 0.64	5.33 ± 0.88	< 0.001	11.46 ± 0.84	9.49 ± 0.85	< 0.001	5.36 ± 1.01	5.39 ± 0.96	0.904	9.31 ± 0.99	9.43 ± 1.03	0.563
ERAb group	6.87 ± 0.52	5.38 ± 0.84	< 0.001	11.50 ± 0.95	9.53 ± 0.81	< 0.001	5.51 ± 1.12	5.49 ± 1.14	0.955	9.48 ± 0.98	9.50 ± 0.96	0.921

*HUD* humeral upward distance of the affected shoulder, *HFD* humeral forward distance of the affected shoulder, *HUD’* humeral upward distance of the contralateral side shoulder, *HFD’* humeral forward distance of the contralateral side shoulder, *IR* internal rotation, *ER* external rotation, *ERAb* external rotation combined abduction

^*^Values were presented as means ± SDs

### Intra- and Inter-observer Agreement

With respect to HFD, HUD, HFD’ and HUD’, the ICCs were greater than 0.7, thus suggesting high intra- and inter-observer reliabilities for these parameters (Table 4).

**Table 4:** Intra- and inter-observer agreement for humeral head position parameters

	Intra-observer agreement			Inter-observer agreement		
	1st evaluation*	2nd evaluation*	ICC	1st observer*	2nd observer*	ICC
HUD 1D	6.907 ± 0.619	6.887 ± 0.597	0.823	6.897 ± 0.576	6.875 ± 0.544	0.874
HFD 1D	11.314 ± 0.862	11.344 ± 0.842	0.915	11.352 ± 0.941	11.346 ± 0.824	0.897
HUD’ 1D	5.327 ± 0.773	5.309 ± 0.862	0.842	5.260 ± 0.814	5.256 ± 0.896	0.832
HFD’ 1D	9.414 ± 0.991	9.424 ± 0.734	0.904	9.507 ± 0.798	9.492 ± 0.795	0.919
HUD 6W	5.307 ± 0.925	5.329 ± 1.060	0.825	5.299 ± 0.995	5.389 ± 1.136	0.792
HFD 6W	9.415 ± 1.173	9.446 ± 1.017	0.781	9.471 ± 0.963	9.509 ± 0.982	0.843
HUD’ 6W	5.309 ± 0.935	5.323 ± 1.001	0.893	5.339 ± 0.980	5.371 ± 1.050	0.767
HFD’ 6W	9.576 ± 1.179	9.528 ± 1.008	0.712	9.558 ± 0.980	9.608 ± 0.979	0.742

*HUD* humeral upward distance of the affected shoulder, *HFD* humeral forward distance of the affected shoulder, *HUD’* humeral upward distance of the contralateral side shoulder, *HFD’* humeral forward distance of the contralateral side shoulder, *ICC* intra-class correlation coefficient

^*^Values were presented as means ± SDs

### Shoulder Joint Functions

Significant variances were observed in different timeframes across each group, evaluated through the constant-Murley score (CMS), American Shoulder and Elbow Surgeons Scale (ASES), and Western Ontario Shoulder Instability Index (WOSI) (*P* < 0.001). Each group displayed progressive improvement over time. Notably, significant differences were found in every functional score among the three shoulder joint immobilization methods at week 6. However, after 3 months, only the WOSI score manifested significant discrepancy among the three groups, while the CMS and ASES scores presented no substantial difference. In addition, no distinctive difference was found in the CMS, ASES, and WOSI scores among the three groups at months 6 and 12 (*P* > 0.05) (Tables 5, 6 and 7).

**Table 5:** Constant-Murley score (CMS)

	6W*	3 M*	6 M*	12 M*	24 M*	P
internal rotation group	37.44 ± 5.05	81.16 ± 4.45	87.27 ± 2.97	92.82 ± 2.47	92.73 ± 2.93	< 0.001
external rotation group	67.77 ± 6.14	79.77 ± 5.08	87.17 ± 3.37	92.04 ± 3.25	91.85 ± 3.35	< 0.001
external rotation combined abduction group	79.78 ± 5.12	81.06 ± 4.39	87.14 ± 2.96	92.72 ± 3.24	93.08 ± 3.58	< 0.001
P						
IR vs ER	< 0.001	0.112	0.857	0.160	0.154	
ER vs ERA	< 0.001	0.162	0.961	0.246	0.058	
IR vs ERA	< 0.001	0.909	0.820	0.856	0.57	

*IR* internal rotation, *ER* external rotation, *ERAb* external rotation combined abduction

^*^Values were presented as means ± SDs

**Table 6:** American Shoulder and Elbow Surgeons Scale (ASES)

	6W*	3 M*	6 M*	12 M*	24 M*	P
internal rotation group	46.94 ± 7.01	87.60 ± 5.61	90.24 ± 2.93	92.55 ± 1.73	92.87 ± 2.02	< 0.001
external rotation group	65.83 ± 5.51	87.89 ± 5.13	89.62 ± 3.21	93.26 ± 2.13	93.42 ± 2.40	< 0.001
external rotation combined abduction group	80.14 ± 5.51	85.92 ± 5.21	89.90 ± 3.22	92.98 ± 2.14	92.84 ± 2.53	< 0.001
P						
IR vs ER	< 0.001	0.772	0.289	0.057	0.209	
ER vs ERA	< 0.001	0.063	0.652	0.471	0.208	
IR vs ERA	< 0.001	0.100	0.564	0.257	0.944	

*IR* internal rotation, *ER* external rotation, *ERAb* external rotation combined abduction

^*^Values were presented as means ± SDs

**Table 7:** Western Ontario Shoulder Instability Index (WOSI)

	6W*	3 M*	6 M*	12 M*	24 M*	P
internal rotation group	914.45 ± 100.08	385.74 ± 79.79	95.63 ± 38.52	69.19 ± 26.89	69.76 ± 27.13	< 0.001
external rotation group	848.26 ± 102.92	327.25 ± 91.09	94.57 ± 37.04	71.66 ± 25.80	72.11 ± 26.03	< 0.001
external rotation combined abduction group	724.4 ± 75.07	276.28 ± 96.73	102.88 ± 34.15	64.26 ± 25.54	64.46 ± 25.41	< 0.001
P						
IR vs ER	< 0.001	< 0.001	0.877	0.615	0.632	
ER vs ERA	< 0.001	0.004	0.253	0.153	0.141	
IR vs ERA	< 0.001	< 0.001	0.301	0.322	0.29	

*IR* internal rotation, *ER* external rotation, *ERAb* external rotation combined abduction

^*^Values were presented as means ± SDs

## Discussion

Prior research has investigated various arm immobilization methods, with resulting findings varying widely among investigators. The technique of immobilizing the arm in an externally rotated position for initial shoulder dislocations first surfaced in the late 1990s [14]. The goal of external rotation is to stress the subscapularis muscle, maintaining the joint capsule and labrum close to the front of the glenoid. Some researchers have discovered that immobilization in external rotation effectively reduces the rate of recurrence [16, 18, 20–24], while others found no significant difference between external and internal rotation methods [9, 25–31]. Consequently, there’s a lack of consensus about whether to use immobilizers to maintain the arm in purely external rotation, or in a combined position of external rotation and abduction.

**Fig. 3:**
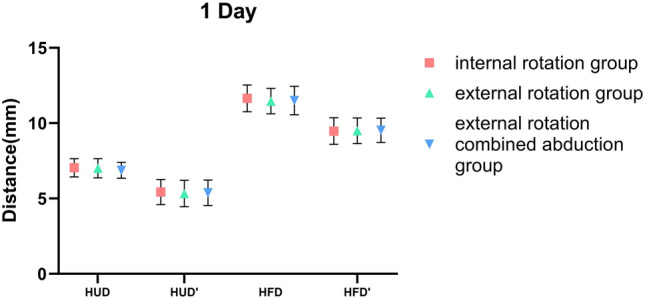
One day after reducing the shoulder joint, we observed the displacement of the humeral head using three immobilization methods

**Fig. 4:**
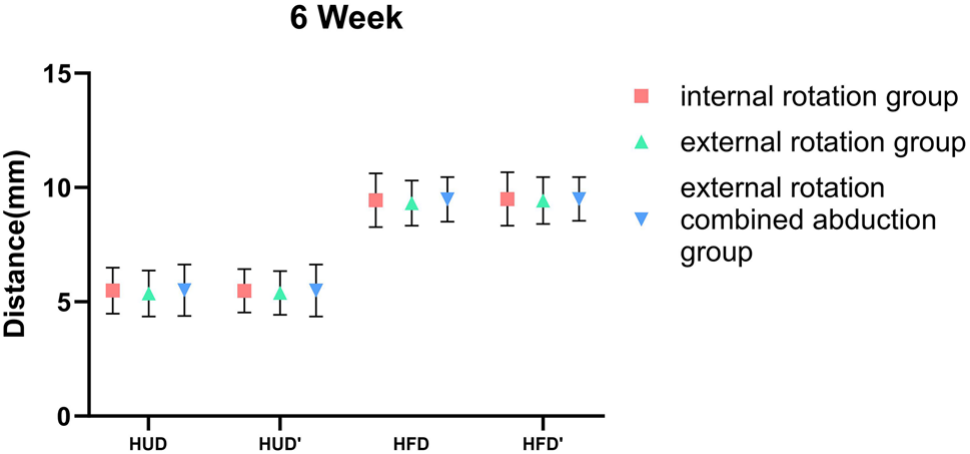
One day following the reduction of the shoulder joint, we examined the shift in the humeral head across three immobilization techniques

**Fig. 5:**
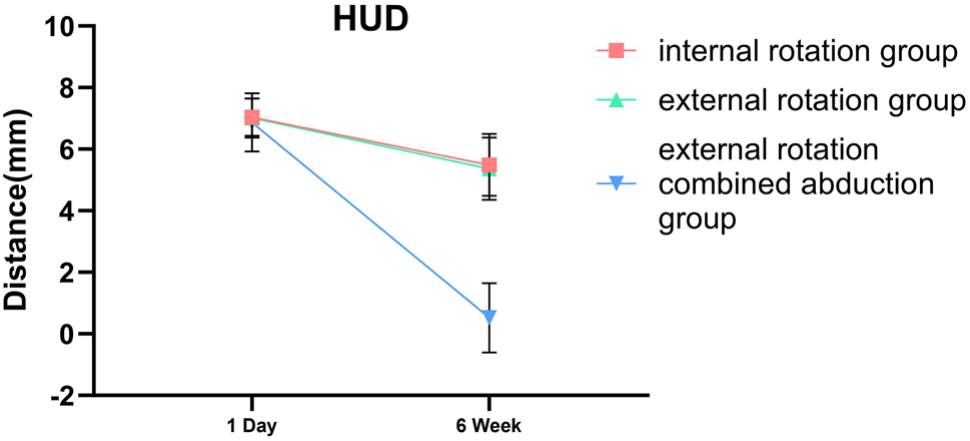
After 6 weeks of reducing the shoulder joint three different immobilization methods were used to restore upward movement in the humeral head

**Fig. 6:**
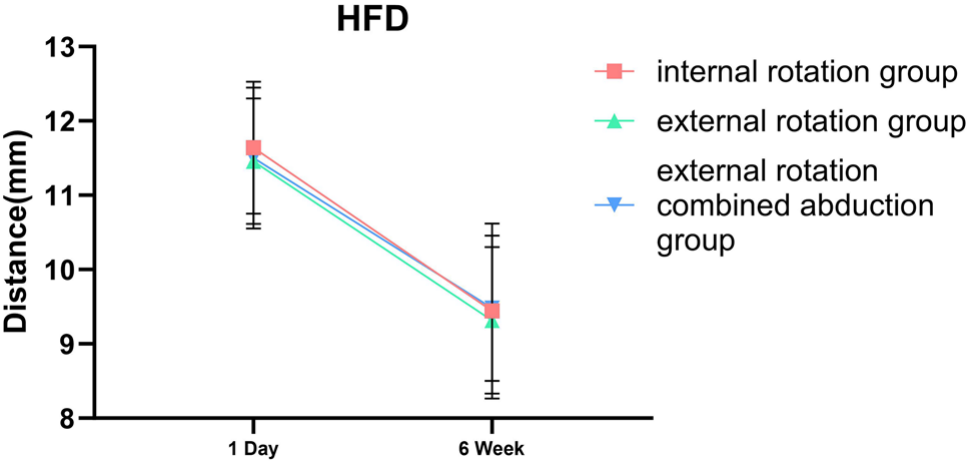
Restoring forward movement in the humeral head after 6 weeks of shoulder joint reduction using three different immobilization methods

This research indicates that irrespective of the immobilization technique used, good outcomes will be attained for anterior shoulder joint dislocations. Post manual reduction of such dislocations, a minor forward and upward shift of the humeral head may still occur (Fig. 3). This observation confirms our previous knowledge surrounding shoulder joint dislocations. When the shoulder dislocates toward the front, it can result in an anterior labral tear and stretching and tearing of the capsule. Younger patients tend to experience an anterior labral tear, while older patients commonly have stretching and tearing of the capsule [32]. In addition to functional scale assessments, We analyzed the quantitative radiographic parameter to objectively assess the subsequent healing status of the joint capsule after success of shoulder dislocation reduction. From the results, if the injury is limited to an isolated capsular tear, without concomitant labral or osseous damage. Residual mild displacement of the humeral head relative to the glenoid following closed reduction of a dislocated shoulder typically resolves spontaneously within 6 weeks, there is no difference among the three internal immobilization methods and capsular healing restores glenohumeral stability for each methods. The humeral head’s position within the shoulder joint essentially mirrors that of the opposing side after 6 week immobilization, as evidenced in Fig. 4, with no marked statistical variance among the groups, as illustrated in Figs. 5 and 6. These findings supply orthopedic physicians with data regarding humeral head displacement following the correction of anterior shoulder joint dislocation. As far as we are aware, this is the first investigation that indirectly gauges humeral head displacement by measuring the gap between the humeral head and the coracoid processes, thus allowing us to observe changes in the humeral head after a manual correction of an anterior shoulder joint dislocation.

**Fig. 7:**
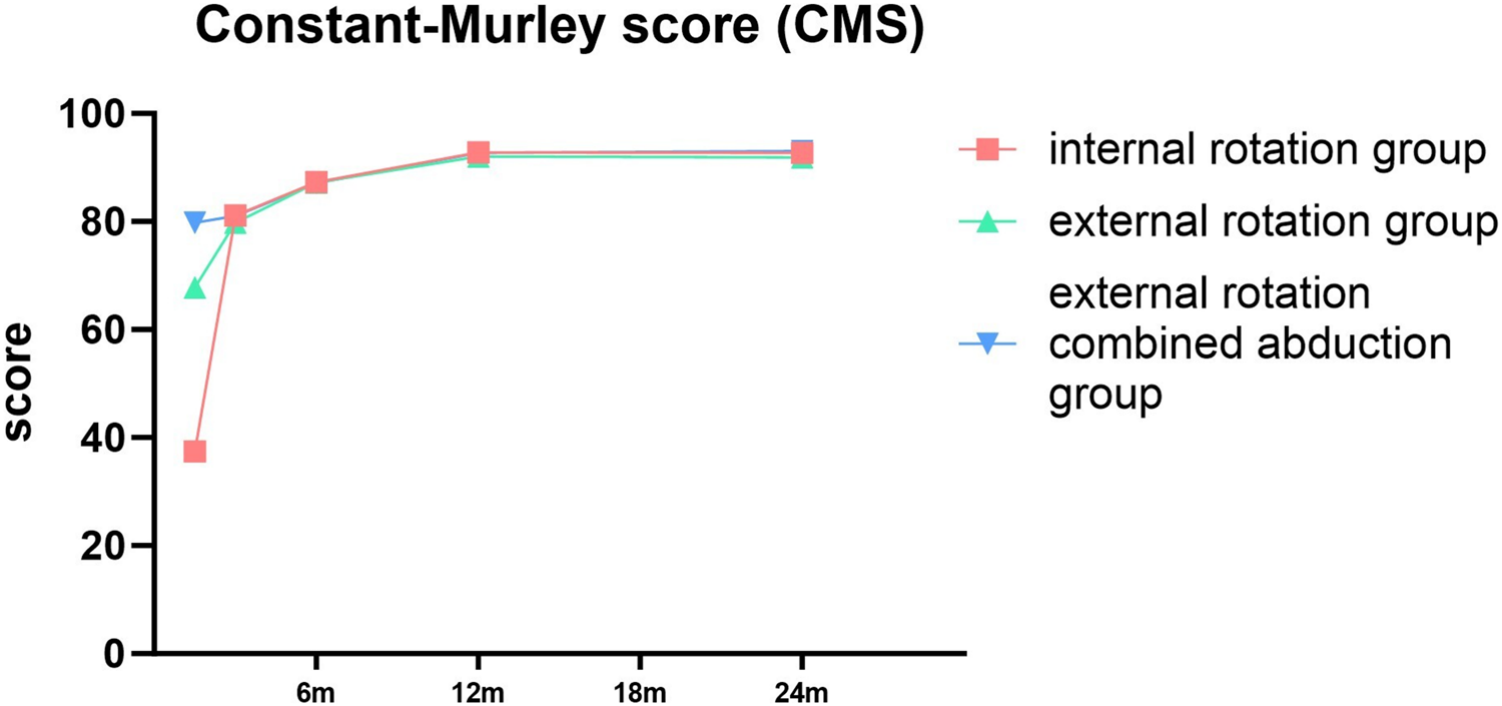
Comparison of constant-Murley score (CMS) for three different immobilization methods following shoulder joint reduction

**Fig. 8:**
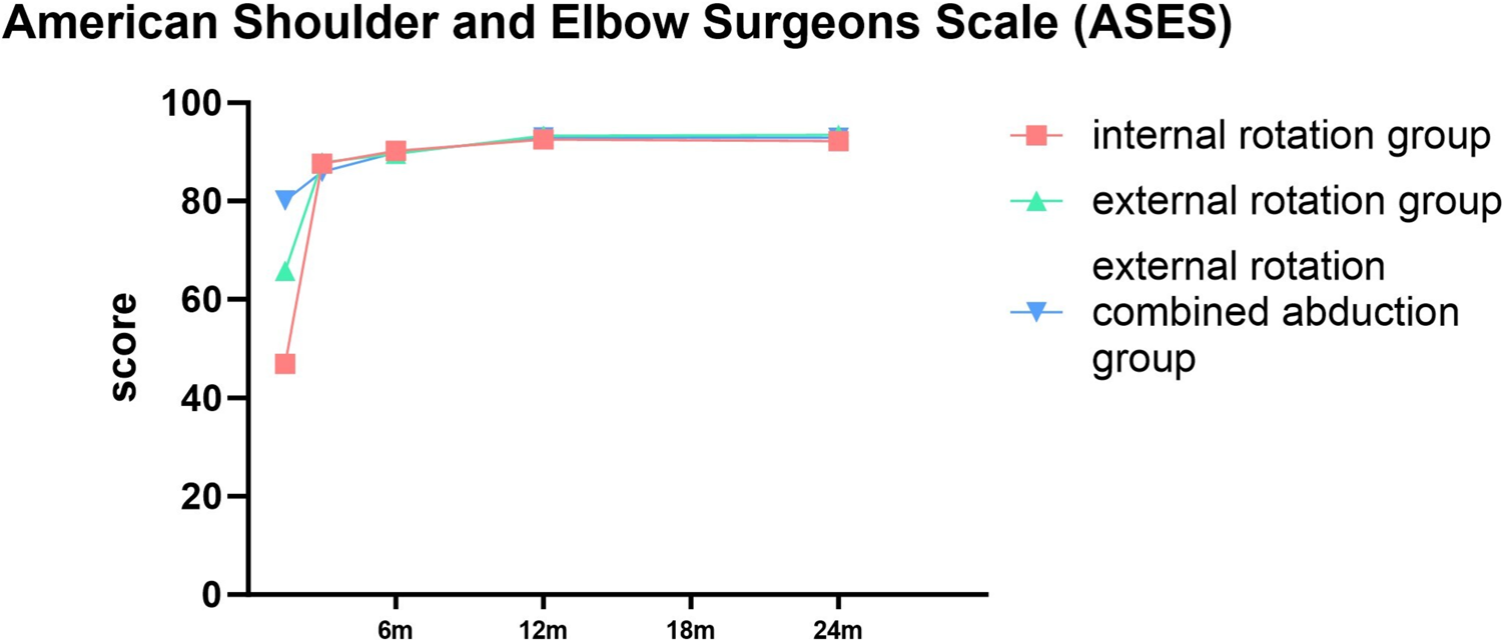
Comparison of American Shoulder and Elbow Surgeons Scale (ASES) for three different immobilization methods following shoulder joint reduction

**Fig. 9:**
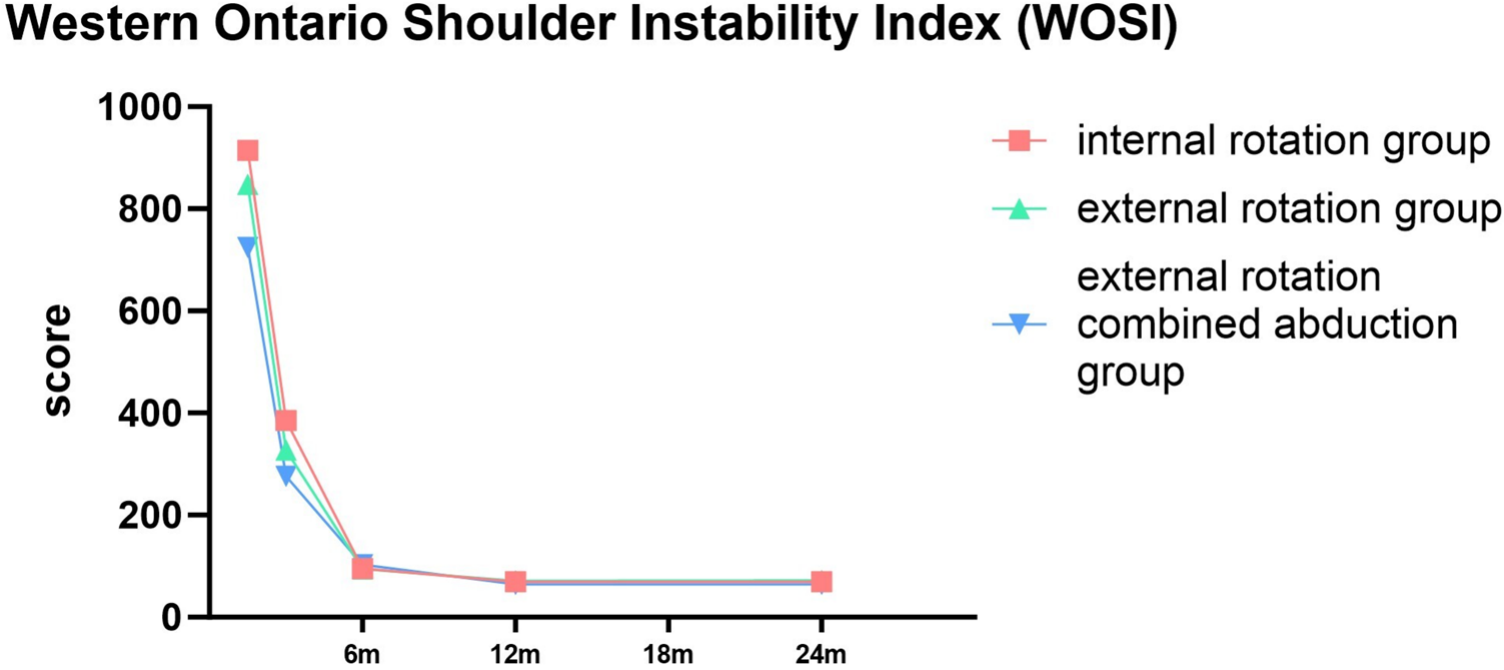
Comparison of Western Ontario Shoulder Instability Index (WOSI for three different immobilization methods following shoulder joint reduction

In terms of shoulder joint function recovery, all three methods of shoulder joint immobilization achieved good results. However, at 6 weeks of shoulder joint reduction, the three groups showed statistical differences. The abduction and external rotation group performed better than the other two groups when the immobilization was just released, this accelerates the reduction of rehabilitation burden, especially in elderly patients with comorbidities. However, over time, the differences between the three groups gradually narrowed. After 3 months of immobilization, there was no significant difference in CMS and ASES between the three groups, while there was still a difference in WOSI, which may be related to the refined scoring system of WOSI, at months 6, 12 and 24 of shoulder joint reduction, there was no statistically significant difference in functional scores among the three groups, and each group achieved satisfactory results (Figs. 7, 8 and 9). While long-term outcomes converge, ERAb’s early functional advantage may reduce socioeconomic burdens (e.g., earlier return to work). For frail elderly, IR remains viable with tailored rehabilitation.

During shoulder joint immobilization, we need to carefully select abduction and external rotation angles. A recent randomized, controlled trial by Itoi et al., found that 30° of abduction and 60° of external rotation is the optimal position to minimize Bankart lesions [18]. Nonetheless, another study by Murray J.C et al., proposed that 15° of external rotation without any abduction is more effective [23]. Thus, we utilize three different methods for shoulder joint immobilization, which include 70° internal rotation (The conventional angle for using forearm sling is 70°), 15° of external rotation, and a further method combining 60° of external rotation with 30° of abduction.

Treating a primary anterior shoulder dislocation with several weeks of immobilization can ideally aid in healing the inflicted soft-tissue damage.[5, 33–35] The duration of immobilization ranging from a few days to 6 weeks in patients experiencing their first dislocation episode, complemented by physiotherapy either during or after this period. For this study, we considered factors, such as the average age of dislocation patients and the prolonged healing time of soft tissue. Therefore, we decided to follow the maximum suggested immobilization time of 6 weeks. Once immobilization was discontinued, the shoulder joint functionality in the internal rotation group was subpar compared to the superior functionality in the external rotation group. This preliminary discrepancy might be due to the more adhesive anterior shoulder joint capsule during internal rotation immobilization compared to the other methods. However, further physical rehabilitation treatment led to no significant difference in the final functional scores among all three groups, displaying satisfactory outcomes across the board.

The occurrence of repeat dislocations is a frequent issue following an initial shoulder joint dislocation. However, research findings on this topic differ significantly. Various scholars have investigated the likelihood of another dislocation happening after the initial shoulder joint is reset, with estimates ranging from as low as 4% to as high as 92% [21, 22, 26, 27, 36, 37]. Two recent systematic reviews, which had varying inclusion criteria and study amounts, looked at prognostic studies. These studies focused on determining risk factors in individuals following non-surgical treatment of an initial traumatic anterior shoulder dislocation. These reviews found recurring instability in patients (re-dislocation or recurrent subluxation) within a scope of 4–74% after at least a year of follow-up [38]; and within a range of 19–88% following a minimum of 2 years of follow-up [39]. However, in our research, we did not observe any recurrence in all study groups during the 2-year follow-up period. This outcome could stem from the demographic composition of our area, which primarily consists of individuals in their middle and later years. Consequently, this leads to an increased average age among the patients involved in the study and a reduced duration for follow-up. As Braun, C et al. suggested in a recent review, a body of research indicates that younger patients and extended follow-up periods are significant risk factors for repeated dislocation of the shoulder joint [9].

This study has several limitations. First, the small sample size can influence the findings. Second, the relatively high average age of the monitored patients could introduce bias. Third, we did not account for individual patient compliance in consistently wearing the shoulder joint immobilization for the entire duration, and we should track compliance through weekly outpatient visits and patient diaries. Finally, the retrospective design introduce bias and lack of randomization, This retrospective design limits causal inference, and this study lacks propensity score matching of baseline features to demonstrate whether physician preferences systematically distort results. Moreover, the retrospective design introduces bias due to non-randomized group allocation based on physician preference. This may have led to uneven distribution of prognostic factors (e.g., younger patients might preferentially receive ERAb). However, Table 1 confirms no significant differences in baseline characteristics (age, sex, BMI, dominant shoulder) among groups (*P* > 0.05), mitigating concerns of major selection bias. Nevertheless, future randomized controlled trials are essential to validate our findings. Going forward, we need to conduct more randomized controlled trials using larger sample sizes and confirming patient compliance. This will allow for a better understanding of how different immobilization methods impact patients across all age groups.

## Conclusion

Abduction coupled with external rotation immobilization shows superior results for functional recovery in a medical setting, the impact of all three methods levels out with continued physical therapy. Therefore, internal rotation, external rotation, along with abduction and external rotation immobilization, are each successful strategies in minimizing postoperative complications from an acute anterior shoulder dislocation, especially in middle-aged and elderly patients. In addition, in patients with good tolerance, we still recommend the external rotation combined abduction immobilization (60° of external rotation and 30° of abduction) approach to promote early recovery of shoulder function.

## Data Availability

The data used to support the findings of this study are included within the article.

## Abbreviations

IR: Internal rotation
ER: External rotation
ERAb: External rotation combined abduction
CMS: Constant-Murley score
ASES: American Shoulder and Elbow Surgeons Scale
WOSI: Western Ontario Shoulder Instability Index
HUD: Humeral upward distance
HUD’: HUD of the contralateral side shoulder
HFD: Humeral forward distance
HFD’: HFD of the contralateral side shoulder
CT: Computed tomography
MRI: Magnetic resonance imaging

## References

[1] Kazár, B., & Relovszky, E. (1969). Prognosis of primary dislocation of the shoulder. *Acta orthopaedica Scandinavica,**40*(2), 216–224. 10.3109/17453676908989501 5365161 10.3109/17453676908989501

[2] Rowe, C. R. (1956). Prognosis in dislocations of the shoulder. *The Journal of Bone and Joint Surgery,**38-a*(5), 957–977. 13367074

[3] Simonet, W. T., & Cofield, R. H. (1984). Prognosis in anterior shoulder dislocation. *The American Journal of Sports Medicine,**12*(1), 19–24. 10.1177/036354658401200103 6703178 10.1177/036354658401200103

[4] Zacchilli, M. A., & Owens, B. D. (2010). Epidemiology of shoulder dislocations presenting to emergency departments in the United States. *The Journal of Bone and Joint Surgery,**92*(3), 542–549. 10.2106/jbjs.I.00450 20194311 10.2106/JBJS.I.00450

[5] Rumian, A., Coffey, D., Fogerty, S., & Hackney, R. (2011). Acute first-time shoulder dislocation. *Orthopaedics and Trauma,**25*(5), 363–368. 10.1016/j.mporth.2011.06.001

[6] Matsen, F. A., 3rd., & Zuckerman, J. D. (1983). Anterior glenohumeral instability. *Clinics in Sports Medicine,**2*(2), 319–338. 9697641

[7] McLaughlin, H. L., & MacLellan, D. I. (1967). Recurrent anterior dislocation of the shoulder. II. A comparative study. *The Journal of Trauma,**7*(2), 191–201. 10.1097/00005373-196703000-00002 6018942 10.1097/00005373-196703000-00002

[8] Smith, R. L., & Brunolli, J. (1990). Shoulder kinesthesia after anterior glenohumeral joint dislocation. *The Journal of Orthopaedic and Sports Physical Therapy,**11*(11), 507–513. 18787264

[9] Braun, C., & McRobert, C. J. (2019). Conservative management following closed reduction of traumatic anterior dislocation of the shoulder. *The Cochrane Database of Systematic Reviews,**5*(5), Cd004962. 10.1002/14651858.CD004962.pub4 31074847 10.1002/14651858.CD004962.pub4 PMC6510174

[10] Kirkley, A., Werstine, R., Ratjek, A., & Griffin, S. (2005). Prospective randomized clinical trial comparing the effectiveness of immediate arthroscopic stabilization versus immobilization and rehabilitation in first traumatic anterior dislocations of the shoulder: Long-term evaluation. *Arthroscopy : the journal of arthroscopic & related surgery : official publication of the Arthroscopy Association of North America and the International Arthroscopy Association.,**21*(1), 55–63. 10.1016/j.arthro.2004.09.018 15650667 10.1016/j.arthro.2004.09.018

[11] Ata, A. M., Tuncer, B., Kara, O., & Başkan, B. (2024). The relationship between kinesiophobia, balance, and upper extremity functions in patients with painful shoulder pathology. *PM & R : The Journal of Injury, Function, and Rehabilitation,**16*(10), 1088–1094. 10.1002/pmrj.13145 10.1002/pmrj.13145 38506398

[12] Ceylan, S., Canli, M., Kuzu, A., & Alkan, H. (2024). Predictors of balance in individuals with adhesive capsulitis: A cross-sectional study. *Ergoterapi ve Rehabilitasyon Dergisi,**12*(2), 97.

[13] Miller, B. S., Sonnabend, D. H., Hatrick, C., O’Leary, S., Goldberg, J., Harper, W., et al. (2004). Should acute anterior dislocations of the shoulder be immobilized in external rotation? A cadaveric study. *Journal of Shoulder and Elbow Surgery,**13*(6), 589–592. 10.1016/j.jse.2004.03.006 15570225 10.1016/j.jse.2004.03.006

[14] Itoi, E., Hatakeyama, Y., Urayama, M., Pradhan, R. L., Kido, T., & Sato, K. (1999). Position of immobilization after dislocation of the shoulder. A cadaveric study. *Journal of Bone and Joint Surgery,**81*(3), 385–390. 10199277

[15] Itoi, E., Sashi, R., Minagawa, H., Shimizu, T., Wakabayashi, I., & Sato, K. (2001). Position of immobilization after dislocation of the glenohumeral joint. A study with use of magnetic resonance imaging. *The Journal of Bone and Joint Surgery,**83*(5), 661–667. 10.2106/00004623-200105000-00003 11379734 10.2106/00004623-200105000-00003

[16] Itoi, E., Hatakeyama, Y., Sato, T., Kido, T., Minagawa, H., Yamamoto, N., et al. (2007). Immobilization in external rotation after shoulder dislocation reduces the risk of recurrence. A randomized controlled trial. *The Journal of Bone and Joint Surgery,**89*(10), 2124–2131. 10.2106/JBJS.F.00654 17908886 10.2106/JBJS.F.00654

[17] Hart, W. J., & Kelly, C. P. (2005). Arthroscopic observation of capsulolabral reduction after shoulder dislocation. *Journal of Shoulder and Elbow Surgery,**14*(2), 134–137. 10.1016/j.jse.2004.07.002 15789005 10.1016/j.jse.2004.07.002

[18] Itoi, E., Kitamura, T., Hitachi, S., Hatta, T., Yamamoto, N., & Sano, H. (2015). Arm abduction provides a better reduction of the Bankart Lesion during immobilization in external rotation after an initial shoulder dislocation. *American Journal of Sports Medicine,**43*(7), 1731–1736. 10.1177/0363546515577782 25855657 10.1177/0363546515577782

[19] Schulz, K. F., Altman, D. G., & Moher, D. (2011). CONSORT 2010 statement: updated guidelines for reporting parallel group randomised trials. *International Journal of Surgery (London, England),**9*(8), 672–677. 10.1016/j.ijsu.2011.09.004 22019563 10.1016/j.ijsu.2011.09.004

[20] Seybold, D., Gekle, C., Fehmer, T., Pennekamp, W., Muhr, G., & Kälicke, T. (2006). Immobilization in external rotation after primary shoulder dislocation. *Chirurg,**77*(9), 821–826. 16775682 10.1007/s00104-006-1181-8

[21] Taskoparan, H., Kilincoglu, V., Tunay, S., Bilgic, S., Yurttas, Y., & Komurcu, M. (2010). Immobilization of the shoulder in external rotation for prevention of recurrence in acute anterior dislocation. *Acta Orthopaedica et Traumatologica Turcica,**44*(4), 278–284. 10.3944/AOTT.2010.2274 21252604 10.3944/AOTT.2010.2274

[22] Heidari, K., Asadollahi, S., Vafaee, R., Barfehei, A., Kamalifar, H., Chaboksavar, Z. A., et al. (2014). Immobilization in external rotation combined with abduction reduces the risk of recurrence after primary anterior shoulder dislocation. *Journal of Shoulder and Elbow Surgery,**23*(6), 759–766. 10.1016/j.jse.2014.01.018 24725898 10.1016/j.jse.2014.01.018

[23] Murray, J. C., Leclerc, A., Balatri, A., & Pelet, S. (2018). Immobilization in external rotation after primary shoulder dislocation reduces the risk of recurrence in young patients. A randomized controlled trial. *Orthopaedics & Traumatology: Surgery & Research,**106*(2), 217–222. 10.1016/j.otsr.2018.10.007 10.1016/j.otsr.2018.10.007 30502026

[24] Zhang, B., Sun, Y., Liang, L., Yu, X., Zhu, L., Chen, S., et al. (2020). Immobilization in external rotation versus internal rotation after shoulder dislocation: A meta-analysis of randomized controlled trials. *Orthopaedics & Traumatology, Surgery & Research : OTSR.,**106*(4), 671–680. 10.1016/j.otsr.2020.03.011 10.1016/j.otsr.2020.03.011 32446811

[25] Finestone, A., Milgrom, C., Radeva-Petrova, D. R., Rath, E., Barchilon, V., Beyth, S., et al. (2009). Bracing in external rotation for traumatic anterior dislocation of the shoulder. *The Journal of Bone and Joint Surgery,**91B*(7), 918–921. 10.1302/0301-620x.91b7.22263 10.1302/0301-620X.91B7.22263 19567857

[26] Liavaag, S., Brox, J. I., Pripp, A. H., Enger, M., Soldal, L. A., & Svenningsen, S. (2011). Immobilization in external rotation after primary shoulder dislocation did not reduce the risk of recurrence: a randomized controlled trial. *The Journal of Bone and Joint Surgery,**93*(10), 897–904. 10.2106/JBJS.J.00416 21498489 10.2106/JBJS.J.00416

[27] Whelan, D. B., Litchfield, R., Wambolt, E., & Dainty, K. N. (2014). Joint Orthopaedic Initiative for National Trials of the S. External rotation immobilization for primary shoulder dislocation: a randomized controlled trial. *Clinical Orthopaedics and Related Research,**472*(8), 2380–2386. 10.1007/s11999-013-3432-6 24385033 10.1007/s11999-013-3432-6 PMC4079853

[28] Chetouani, M., Ropars, M., Marin, F., Huten, D., Duvauferrier, R., & Thomazeau, H. (2010). Is MRI useful to assess labral reduction following acute anterior shoulder dislocation? *Orthopaedics & Traumatology, Surgery & Research: OTSR,**96*(3), 203–207. 10.1016/j.otsr.2009.12.004 10.1016/j.otsr.2009.12.004 20488136

[29] Paterson, W. H., Throckmorton, T. W., Koester, M., Azar, F. M., & Kuhn, J. E. (2010). Position and duration of immobilization after primary anterior shoulder dislocation: a systematic review and meta-analysis of the literature. *The Journal of Bone and Joint Surgery,**92*(18), 2924–2933. 10.2106/JBJS.J.00631 21159993 10.2106/JBJS.J.00631

[30] Liu, A., Xue, X., Chen, Y., Bi, F., & Yan, S. (2014). The external rotation immobilisation does not reduce recurrence rates or improve quality of life after primary anterior shoulder dislocation: a systematic review and meta-analysis. *Injury,**45*(12), 1842–1847. 10.1016/j.injury.2014.06.005 25150749 10.1016/j.injury.2014.06.005

[31] Whelan, D. B., Kletke, S. N., Schemitsch, G., & Chahal, J. (2016). Immobilization in external rotation versus internal rotation after primary anterior shoulder dislocation: A meta-analysis of randomized controlled trials. *The American Journal of Sports Medicine,**44*(2), 521–532. 10.1177/0363546515585119 26116355 10.1177/0363546515585119

[32] Wen, D. Y. (1999). Current concepts in the treatment of anterior shoulder dislocations. *American Journal of Emergency Medicine,**17*(4), 401–407. 10452444 10.1016/s0735-6757(99)90097-9

[33] Youm T, Takemoto R, Park BK-H. Acute management of shoulder dislocations. J Am Acad Orthop Surg. 2014;22(12):761–71.10.5435/JAAOS-22-12-761 10.5435/JAAOS-22-12-761 25425611

[34] Khiami, F., Gerometta, A., & Loriaut, P. (2015). Management of recent first-time anterior shoulder dislocations. *Orthopaedics & Traumatology: Surgery & Research,**101*(1), S51–S57. 10.1016/j.otsr.2014.06.027 10.1016/j.otsr.2014.06.027 25596982

[35] Robinson, C. M., Kelly, M., & Wakefield, A. E. (2002). Redislocation of the shoulder during the first six weeks after a primary anterior dislocation: Risk factors and results of treatment. *The Journal of Bone and Joint Surgery American Volume.,**84*(9), 1552–1559. 12208911 10.2106/00004623-200209000-00007

[36] Arciero, R. A., Wheeler, J. H., Ryan, J. B., & McBride, J. T. (1994). Arthroscopic Bankart repair versus nonoperative treatment for acute, initial anterior shoulder dislocations. *The American Journal of Sports Medicine.,**22*(5), 589–594. 7810780 10.1177/036354659402200504

[37] Chan, S. K., Bentick, K. R., Kuiper, J. H., & Kelly, C. P. (2019). External rotation bracing for first-time anterior dislocation of the shoulder: A discontinued randomised controlled trial comparing external rotation bracing with conventional sling. *Shoulder Elbow,**11*(4), 256–264. 10.1177/1758573218768521 31316586 10.1177/1758573218768521 PMC6620796

[38] Olds, M., Ellis, R., Donaldson, K., Parmar, P., & Kersten, P. (2015). Risk factors which predispose first-time traumatic anterior shoulder dislocations to recurrent instability in adults: A systematic review and meta-analysis. *British Journal of Sports Medicine,**49*(14), 913–922. 10.1136/bjsports-2014-094342 25900943 10.1136/bjsports-2014-094342 PMC4687692

[39] Wasserstein, D. N., Sheth, U., Colbenson, K., Henry, P. D. G., Chahal, J., Dwyer, T., et al. (2016). The true recurrence rate and factors predicting recurrent instability after nonsurgical management of traumatic primary anterior shoulder dislocation: A systematic review. *Arthroscopy : the journal of arthroscopic & related surgery : official publication of the Arthroscopy Association of North America and the International Arthroscopy Association.,**32*(12), 2616–2625. 10.1016/j.arthro.2016.05.039 27487737 10.1016/j.arthro.2016.05.039

